# Multidrug‐resistant *Enterococcus faecium* strains enter the Norwegian marine environment through treated sewage

**DOI:** 10.1002/mbo3.1397

**Published:** 2024-03-05

**Authors:** Vera Radisic, Didrik H. Grevskott, Nadja Junghardt, Lise Øvreås, Nachiket P. Marathe

**Affiliations:** ^1^ Department of Contaminants and Biohazards Institute of Marine Research (IMR) Bergen Norway; ^2^ Department of Biological Sciences University of Bergen (UiB) Bergen Norway

**Keywords:** CC17, *Enterococcus faecium*, last resort antibiotic, MDR strain, Norway, sewage surveillance

## Abstract

This study aimed to understand the antibiotic resistance prevalence among *Enterococcus* spp. from raw and treated sewage in Bergen city, Norway. In total, 517 *Enterococcus* spp. isolates were obtained from raw and treated sewage from five sewage treatment plants (STPs) over three sampling occasions, with *Enterococcus faecium* as the most prevalent (*n* = 492) species. *E. faecium* strains (*n* = 307) obtained from the influent samples, showed the highest resistance against quinupristin/dalfopristin (67.8%). We observed reduced susceptibility to erythromycin (30.6%) and tetracycline (6.2%) in these strains. *E. faecium* strains (*n* = 185) obtained from the effluent samples showed highest resistance against quinupristin/dalfopristin (68.1%) and reduced susceptibility to erythromycin (24.9%) and tetracycline (8.6%). We did not detect resistance against last‐resort antibiotics, such as linezolid, vancomycin, and tigecycline in any of the strains. Multidrug‐resistant (MDR) *E. faecium* strains were detected in both influent (2.3%) and effluent (2.2%) samples. Whole genome sequencing of the *Enterococcus* spp. strains (*n* = 25) showed the presence of several antibiotic resistance genes, conferring resistance against aminoglycosides, tetracyclines, and macrolides, as well as several virulence genes and plasmid replicons. Two sequenced MDR strains from the effluents belonged to the hospital‐associated clonal complex 17 and carried multiple virulence genes. Our study demonstrates that clinically relevant MDR *Enterococcus* spp. strains are entering the marine environment through treated sewage.

## Graphical abstract

This study investigated the prevalence of antibiotic resistance in *Enterococcus* spp. from raw and treated sewage in Bergen, Norway. We analyzed 307 *Enterococcus faecium* isolates from raw sewage and 185 *E. faecium* isolates from treated sewage for antibiotic sensitivity and sequenced selected isolates (*n* = 25). This study found that multidrug‐resistant *E. faecium* clones from clonal complex 17 are entering the marine environment through treated sewage.

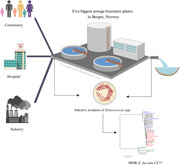

## 1 INTRODUCTION

*Enterococcus* spp. consists of Gram‐positive, opportunistic pathogens that are commonly found in the intestinal microbiota of healthy humans and animals (Byappanahalli et al., 2012; Iversen et al., 2002). *Enterococcus* spp. are a major cause of hospital‐acquired infections (HAI) and are ranked second as a cause of bloodstream‐ and urinary tract infections in the European Union/European Economic Area (EU/EEA) (ECDC, 2023; Viswanath et al., 2022). *Enterococcus faecium* (around 90%) and *Enterococcus faecalis* (around 5%–10%) are responsible for most enterococcal infections in humans (Simonsen, 2003; Top et al., 2008). Other species belonging to this genus, like the species *E. durans, E. gallinarum*, and *E. casseliflavus*, can also cause infections in humans (Arias & Murray, 2012; Liassine et al., 1998; Murray, 1990; Rahimi et al., 2007).

*E. faecium* is a part of the ESKAPE pathogens (consisting of *E. faecium*, *Staphylococcus aureus*, *Klebsiella pneumoniae*, *Acinetobacter baumannii*, *Pseudomonas aeruginosa*, and *Enterobacter* spp.), which represent pathogens that are leading causes of nosocomial infections worldwide (Santajit & Indrawattana, 2016). Vancomycin‐resistant *E. faecium* (VRE) is of special concern and poses a growing health problem due to limited available treatment options (Hayakawa et al., 2014). Linezolid, daptomycin, and tigecycline are last‐resort antibiotics used for the treatment of such infections (Diaz et al., 2014; Klare et al., 2015; Werner et al., 2012). Consequently, the World Health Organization (WHO) has listed this pathogen as a high priority for finding new treatments (WHO, 2023). Increasing trends of VRE, ranging from 11.6% in 2016 to 16.8% in 2020, are observed in the EU/EEA (ECDC, 2022). In Norway, only one VRE strain with clinical importance (*E. faecium* carrying *vanB*) was obtained from blood culture, while no linezolid‐resistant strains were observed in the Norwegian monitoring report (NORM/NORM‐VET) in 2021 (NORM/NORM‐VET, 2023), suggesting a low prevalence of vancomycin resistance in *Enterococcus* spp. in clinics in Norway.

The prevalence of resistant *Enterococcus* spp. within the population in Norway is largely unknown. Surveillance using a sewage‐based approach has shown to be promising for understanding antibiotic resistance in different pathogens within a studied community (Grevskott et al., 2021; Radisic et al., 2023). This study aimed at understanding the frequency of antibiotic resistance among *Enterococcus* spp. present within the population using sewage‐based surveillance. Further, we investigated the antibiotic resistance prevalence in *Enterococcus* spp. entering the marine environments through treated effluent.

## 2 MATERIALS AND METHODS

### 2.1 Sewage sample collection

The details about the collection of sewage samples used in this study have previously been described in Radisic et al. (2023). Briefly: on three different time points (1 March, 3 May, and 5 July 2021) the sewage samples (both influents and effluents) were collected from five sewage treatment plants (STPs) (Flesland, 152,000 citizens; Holen, 132,000 citizens; Knappen, 63,000 citizens; Kvernevik, 56,000 citizens; and Ytre‐Sandviken, 44,000 citizens) located in Bergen, Norway (Radisic et al., 2023), using a 24‐h time sampler. The complete overview of the different STPs, capacity, and type of treatment are presented in Table S1.

### 2.2 Isolation and identification of isolates

The sewage samples were serially diluted (10‐fold) with sterile saline (0.85% NaCl) and plated on Slanetz and Bartley media (Thermo Fisher Scientific) within 6 h of collection. The plates were aerobically incubated at 44°C for 48 h. The number of presumptive *Enterococcus* spp. was estimated by counting maroon and/or pink colonies. Up to 30 isolates were randomly picked from the Slanetz and Bartley plates for each sample and transferred to Mueller–Hinton broth (Sigma‐Aldrich), followed by aerobic incubation at 44°C for another 48 h. Afterward, 20% glycerol was added, and the samples were stored at −80°C till further use.

### 2.3 Antibiotic susceptibility testing (AST)

From the glycerol stocks, the strains were re‐streaked onto Slanetz and Bartley plates and incubated at 44°C for 48 h, followed by identification using MALDI‐TOF MS (Bruker Daltonics). The resistance profiles of 517 *Enterococcus* spp. strains were determined against 12 antibiotics using a broth microdilution assay with Sensititre® EUVENC plates (Thermo Fisher Scientific) following the manufacturer's protocol. Each strain was tested for resistance and/or reduced susceptibility against 12 antibiotics (Table S2). The plates were incubated at 37°C for 24 h. The strains were defined as susceptible and/or resistant according to the European Committee on Antimicrobial Susceptibility Testing (EUCAST) clinical breakpoints tables v.13.0 (EUCAST, 2023). For gentamicin, the minimum inhibitory concentration (MIC) breakpoint for high‐level aminoglycoside resistance (>128 mg/L) was used when defining resistance. For antibiotics with clinical breakpoints not available in EUCAST, epidemiological cut‐off (ECOFF) values were used for defining reduced susceptibility compared to wild‐type (WT) *Enterococcus* spp. strains with MIC above the ECOFF were classified as strains with reduced susceptibility. No ECOFF values were available for *E. durans* and *E. casseliflavus. E. faecium* CCUG 542T and *E. faecalis* CCUG 19916T were used as controls for the Sensititre® plates.

### 2.4 DNA extraction and sequencing

Whole genome sequencing was performed, for 24 presumptive *E. faecium* strains and one *E. faecalis* strain, selected on the basis of phenotypic resistance profiles. The strains were re‐streaked on Mueller–Hinton agar (Oxoid) at 44°C for 48 h. Genomic DNA was processed and sequenced as described in Radisic et al. (2023). Briefly: sequencing libraries were prepared using Nextera DNA Flex Library Prep kit (Illumina) and sequencing was performed using an Illumina MiSeq‐based platform (Illumina), with 2 × 300 bp chemistry.

### 2.5 Genome assembly and sequencing analysis

Genome assembly and annotation was accomplished as previously described in Radisic et al. (2020, 2023). Briefly, the raw reads generated by the Illumina MiSeq sequencing were quality‐filtered and assembled using SPAdes (v.3.13.0) (Bankevich et al., 2012). Annotation was performed with the NCBI Prokaryotic Genome Annotation Pipeline (Tatusova et al., 2013). Antibiotic resistance genes (ARGs) were identified using CARD v.3.1.4 (Alcock et al., 2019) and ResFinder v.4.1 (Bortolaia et al., 2020). Plasmids were identified with PlasmidFinder v.2.1 (Carattoli et al., 2014). VFanalyzer at the VFDB (Liu et al., 2019) were used to check for virulence genes. Sequence types (STs) and clonal complexes (CCs) were identified with the PubMLST database (https://pubmlst.org/bigsdb?db=pubmlst_efaecium_seqdef and https://pubmlst.org/bigsdb?db=pubmlst_efaecalis_seqdef). Identification of sequenced strains was performed, using the average nucleotide identity based on BLAST (ANIb) analysis on the JWSpecies server (http://jspecies.ribohost.com/jspeciesws/) with recommended cut‐off values of >95% identity (Chun & Rainey, 2014) for species assignment.

### 2.6 Core genome‐based phylogeny of the strains

A phylogenetic tree was constructed based on the core genomes of selected sequenced *E. faecium* strains (*n* = 10) belonging to CC17 to check the clade (A1, A2, and/or B) our strains are clustering with. Strain ATCC700221 (GenBank accession no. GCA_001594345.1) was used as a reference strain for clade A1, while strain NCTC7174 (GCA_900637035.1) was used as a reference for clade A2. The assembled genomes were analyzed with CSI Phylogeny v.1.4 (https://cge.food.dtu.dk/services/CSIPhylogeny/), using the parameters previously described (Radisic et al., 2023). All strains used for constructing the phylogenetic tree are presented in Table S3.

## 3 RESULTS

### 3.1 *Enterococcus* spp. isolates from sewage

A higher number of the presumptive *Enterococcus* spp. were obtained from the influent samples (*n* = 92; *n* = 99; *n* = 125) compared to the effluent samples (*n* = 41; *n* = 90; *n* = 70) for all five STPs during the three sampling points. From all samples, a total of 627 isolates were obtained and identified using MALDI‐TOF MS. Out of 627 isolates, 517 isolates (82.5%) were identified as *Enterococcus* spp. with *E. faecium* as most prevalent (*n* = 492) followed by *E. faecalis* (*n* = 13), *E. hirae* (*n* = 7), *E. durans* (*n* = 3), and *E. casseliflavus* (*n* = 2). Isolates identified as other genera like *Streptococcus infantarius* (*n* = 88), *S. equinus* (*n* = 12), and *S. lutetiensis* (*n* = 10), were excluded from further analysis.

### 3.2 Resistance rates in *E. faecium* strains

In total, 307 presumptive *E. faecium* strains were isolated from the influent samples, and 185 presumptive *E. faecium* strains were isolated from the effluent samples. Strains obtained from the influents showed the highest resistance against quinupristin/dalfopristin (67.8%) and ciprofloxacin (4.2%). Reduced susceptibility to erythromycin and tetracycline was observed in 30.6% and 6.2% of the strains, respectively. Strains obtained from the effluent samples also showed the highest resistance against quinupristin/dalfopristin (68.1%), followed by ciprofloxacin (3.2%), high‐level gentamicin (1.1%) and ampicillin (1.1%). Additionally, *E. faecium* strains recovered from the effluent samples also showed reduced susceptibility to erythromycin (24.9%) and tetracycline (8.6%). During the three sampling occasions, no resistance against vancomycin, teicoplanin, daptomycin, tigecycline, linezolid, or chloramphenicol was observed (Table S2). From a total of 492 presumptive *E. faecium* strains, 2.2% were classified as multidrug‐resistant (MDR), showing resistance against at least one antibiotic belonging to three or more classes. Two of these strains (E4‐112 and E5‐100) detected in the treated effluent, showed resistance against ampicillin, ciprofloxacin, and high‐level gentamicin resistance.

### 3.3 AST of other *Enterococcus* spp. strains

From both influent and treated effluent during the three sampling occasions, 25 isolates were identified as other *Enterococcus* spp. For *E. faecalis*, reduced susceptibility to tetracycline (23.1%, *n* = 3) and erythromycin (7.7%, *n* = 1) was observed. We further observed resistance against gentamicin in 7.7% (*n* = 1) of the strains. Resistance and/or reduced susceptibility for *E. hirae, E. durans*, or *E. casseliflavus* was not determined due to a lack of defined clinical breakpoints and/or no available ECOFF values.

### 3.4 Genome sequence and STs of sequenced *Enterococcus* spp. strains

The overview of the assembly statistics of the 25 sequenced genomes is presented in Table S4. Four strains (E4‐185, E5‐9, E5‐80, and E5‐162), which were identified as *E. faecium* by MALDI‐TOF MS, were after sequencing re‐identified as *E. lactis* (ANIb values ≥ 97.75%) and not as *E. faecium*. The most prevalent ST among the sequenced *E. faecium* strains was ST32 (20%, *n* = 4), while all sequenced *E. lactis* strains belonged to different STs. *E. faecalis* strain belonged to ST227. Several strains (E4‐85, E4‐150, E4‐152, E4‐162, E4‐185, E4‐223, E5‐37, E5‐79, E5‐103, E5‐112, E5‐162, and E5‐209) were not assigned to any ST and/or CC using the PubMLST database.

### 3.5 Single nucleotide polymorphism (SNP)‐based phylogeny of CC17 strains

**Figure 1:**
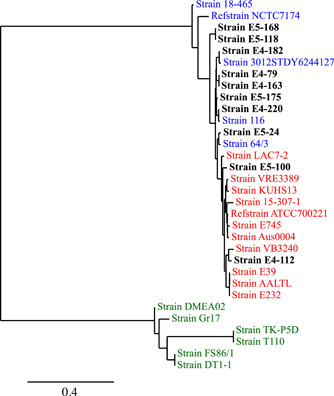
Phylogenetic tree based on the whole genome sequences of *Enterococcus faecium* strains. Strains belonging to clade A1 are highlighted in red, while strains belonging to clade A2 and B are highlighted in blue and green, respectively. Our strains (E4‐79, E4‐112, E4‐163, E4‐182, E4‐220, E5‐24, E5‐100, E5‐119, E5‐168, and E5‐175) are highlighted in bold. Two strains ATCC700221 (GenBank accession no. GCA_001594345.1) and NCTC7174 (GCA_900637035.1) were used as reference strains for clade A1 and A2, respectively.

The most prevalent CC among the sequenced *E. faecium* strains was CC17 (*n* = 10). The SNP‐based phylogeny showed that most of these strains (E4‐79, E4‐163, E4‐182, E4‐220, E5‐24, E5‐119, E5‐168, and E5‐175) belonging to CC17 clustered with clade A2, representing animal derived strains (Gao et al., 2018). Two MDR strains (E4‐112 and E5‐100), showing high‐level gentamicin resistance as well as ampicillin and ciprofloxacin resistance, clustered with clinical strains belonging to clade A1 (Figure 1).

### 3.6 Clinically important ARGs, virulence genes, and plasmid replicons present in sequenced *Enterococcus* spp. strains

All sequenced *E. faecium* strains (*n* = 20) carried the known intrinsic ARG, *aac(6′)‐li* while ARGs like *msr*(C) and *aph(3′)‐IIIa* were detected in 95% and 15% of the strains, respectively. We also detected ARGs such as *msr*(A) and *msr*(B), which have previously been detected in *Enterococcus* spp. (Hummel et al., 2007; Roberts, 2008). in all sequenced strains (Table 1). Several acquired clinically important ARGs were detected in the sequenced *Enterococcus* spp. strains (*n* = 25), conferring resistance against aminoglycosides (bifunctional *acc(6′)‐le/aph(2″)‐la*, *aph(2″)‐la*, *ant(6)‐Ia*, and *ant(9)*), tetracyclines (*tet*(L) and *tet*(M)), and macrolides (*erm*(B) and *erm*(T)) (Table 1). Two strains (E4‐112 and E5‐100) showing high ciprofloxacin resistance with MIC > 16 mg/L carried known murtations in *gyrA* (p.S83Y) and *parC* (p.S80I) (Leavis, Willems, et al., 2006).

**Table 1:** List of antibiotic resistance genes (ARGs) and plasmid replicons detected in the 25 sequenced *Enterococcus* spp. strains.

Strain	Species	Sample type	ARGs	Plasmid replicons
E4‐79	*E. faecium*	Influent	*msr*(A), *msr*(B), *msr*(C), *erm*(T), *tet*(L), *tet*(M), *aac(6)‐Ii*	rep2, repUS12, repUS43
E4‐85	*E. faecium*	Influent	*msr*(A), *msr*(B), *msr*(C), *aac(6′)‐Ii*	rep1, repUS15
E4‐112	*E. faecium*	Effluent	*msr*(A), *msr*(B), *msr*(C), *tet*(M), *aac(6′)‐Ii*, bifunctional *aac(6′)‐Ie/aph(2″)‐Ia*	rep2, rep29, repUS15
E4‐150	*E. faecium*	Influent	*msr*(A), *msr*(B), *msr*(C), *aac(6′)‐Ii*	rep1, rep2, rep18a, repUS15,
E4‐152	*E. faecium*	Influent	*msr*(A), *msr*(B), *msr*(C), *aac(6′)‐Ii*	‐
E4‐162	*E. faecium*	Influent	*msr*(A), *msr*(B), *msr*(C), *erm*(B), *lnu*(B), *lsa*(E), *tet*(M), *aac(6′)‐Ii*, *aph(3′)‐IIIa*, *ant(6)‐Ia*, *ant(9)*	rep1, repUS15, repUS43
E4‐163	*E. faecium*	Influent	*msr*(A), *msr*(B), *msr*(C), *erm*(T), *tet*(L), *tet*(M), *aac(6′)‐Ii*	rep2, repUS12, repUS43
E4‐182	*E. faecium*	Effluent	*msr*(A), *msr*(B), *msr*(C), *erm*(B), *lnu*(B), *lsa*(E), *tet*(L), *tet*(M), *aac(6′)‐Ii*, *aph(3′)‐IIIa*, *ant(9)*, aminoglycoside 6‐adenylyltransferase	rep2, repUS43
E4‐185	*E. lactis*	Effluent	*msr*(A), *msr*(B), *msr*(C), *erm*(T), *tet*(L), *tet*(M), *aac(6′)‐Ii*	rep1, rep2, repUS12, repUS15, repUS43
E4‐208	*E. faecium*	Effluent	*msr*(A), *msr*(B), *msr*(C), *tet*(M), *aac(6′)‐Ii*	rep1, repUS15, repUS42
E4‐220	*E. faecium*	Influent	*msr*(A), *msr*(B), *msr*(C), *aac(6′)‐Ii*	‐
E4‐223	*E. faecium*	Influent	*msr*(A), *msr*(B), *msr*(C), *aac(6′)‐Ii*	rep1, repUS15
E5‐9	*E. lactis*	Influent	*msr*(A), *msr*(B), *msr*(C), *tet*(L), *aac(6′)‐Ii*, *str*	rep22, repUS15
E5‐24	*E. faecium*	Influent	*msr*(A), *msr*(B), *msr*(C), *erm*(T), *tet*(L), *tet*(M), *aac(6′)‐Ii*	repUS12, repUS43
E5‐37	*E. faecium*	Effluent	*msr*(A), *msr*(B), *msr*(C), *tet*(M), *aac(6′)‐Ii*	repUS15, repUS43
E5‐79	*E. faecium*	Influent	*msr*(A), *msr*(B), *msr*(C), *aac(6′)‐Ii*	‐
E5‐80	*E. lactis*	Influent	*msr*(A), *msr*(B), *msr*(C), *tet*(L), *tet*(M), *aac(6′)‐Ii*, *str*	rep1, rep22, repUS15
E5‐100	*E. faecium*	Effluent	*msr*(A), *msr*(B), *erm*(B), *aac(6′)‐Ii*, *aph(2″)‐Ia*, *aph(3′)‐IIIa*, *ant(6)‐Ia*, *catA‐8*	rep2, repUS15
E5‐103	*E. faecalis*	Influent	*msr*(A), *msr*(B), *tet*(M), *lsa*(A)	repUS43
E5‐112	*E. faecium*	Influent	*msr*(A), *msr*(B), *msr*(C), *aac(6′)‐Ii*	‐
E5‐118	*E. faecium*	Influent	*msr*(A), *msr*(B), *msr*(C), *aac(6′)‐Ii*	repUS15
E5‐162	*E. lactis*	Influent	*msr*(A), *msr*(B), *msr*(C), *tet*(L), *tet*(M), *aac(6′)‐Ii*, *ant(6)‐Ia*, *catA*	repUS12, repUS15, repUS43
E5‐168	*E. faecium*	Influent	*msr*(A), *msr*(B), *msr*(C), *aac(6′)‐li*	repUS15
E5‐175	*E. faecium*	Influent	*msr*(A), *msr*(B), *msr*(Crep1′), *tet*(L), *tet*(M), *aac(6′)‐li*	rep2, repUS12, repUS43
E5‐209	*E. faecium*	Influent	*msr*(A), *msr*(B), *msr*(C),*aac(6′)‐li*	rep1

Plasmid replicons were detected in most sequenced *E. faecium* strains (84%), with plasmid replicon type repUS15 as most common (50%), followed by repUS43 (35%) and rep2 (35%). More than one plasmid replicon type was observed in 68% of the sequenced *Enterococcus* spp. strains. *E. faecalis* carried the plasmid replicon type repUS43, whereas repUS15 was the most frequently observed plasmid replicon type in *E. lactis* (100%) (Table 1).

All sequenced *E. faecium* strains carried multiple virulence factors involved in antiphagocytosis (capsule; *cpsA/uppS, cpsB/cdsA*) and surface proteins (*lgt*), as well as virulence factors involved in biofilm formation (*bopD*) (Qin et al., 2012). In addition to these, virulence factors involved in adherence like the virulence factors EfaA (100%) (Bourgogne et al., 2006), Ebp pili (95%), Acm (80%), Scm (20%), SgrA (15%), and EcbA (10%) were also detected in the sequenced strains (Hendrickx et al., 2009; Qin et al., 2012). The *E. lactis* strains (*n* = 4) carried virulence genes involved in adherence, antiphagocytosis, biofilm formation (Oxaran et al., 2012), enzymes (hyaluronidase and serine‐threonine phosphatase from *Listeria*) (Stedman et al., 2020) and surface protein anchoring (*lgt*). *E. faecalis* carried various virulence factors involved in adherence (Ace, Ebp pili, EfaA) (Golob et al., 2019; Kayaoglu & Ørstavik, 2004), antiphagocytosis (capsule; *cpsA/uppS*, *cpsB/cdsA*, and *cpsC*) (Hufnagel et al., 2004), biofilm formation (*bopD*, *fsrA*, *fsrB*, and *fsrC*), enzymes (*gelE* and *sprE*) (Engelbert et al., 2004; Hancock & Perego, 2004), and immune evasion (capsule from *Staphylococcus*). The complete overview of the different virulence genes is presented in Table S5.

## 4 DISCUSSION

To the best of our knowledge, this is the first study performing population‐based surveillance of antibiotic resistance in *Enterococcus* spp. on a city scale in Norway. Although we did not detect any resistance against last‐resort antibiotics, such as linezolid, vancomycin, and tigecycline, we did detect MDR *Enterococcus* spp. strains in our study. Our findings are in accordance with the prevalence of resistance against last‐resort antibiotics reported from the clinics and the animal sector in Norway (NORM/NORM‐VET, 2022).

Quinupristin/dalfopristin is often used to treat MDR *E. faecium* infections. Quinupristin/dalfopristin resistance in clinical *E. faecium* strains has been reported as a concern in several countries (Wang et al., 2016). From a total of 307 presumptive *E. faecium* strains obtained from the influent samples during the three sampling points, 67.8% of these strains showed phenotypic resistance against quinupristin/dalfopristin. A similar resistance rate was observed against quinupristin/dalfopristin (68.1%) in 185 presumptive *E. faecium* strains obtained from effluents. Our results are in accordance with the resistance prevalence detected in *E. faecium* from fattening pigs in Norway, where 67.3% of the strains were resistant against quinupristin/dalfopristin (NORM/NORM‐VET, 2022). Our results are also in accordance with the high resistance prevalence against quinupristin/dalfopristin in *E. faecium* from healthy food‐producing animals in Europe (de Jong et al., 2019). Quinupristin/dalfopristin resistance in *E. faecium* can be achieved through several different mechanisms, including the presence of acquired ARGs like *vat*(D), *vat*(E), *vgb*(A), *vgb*(B), and *eat*(A), as well as the presence of the intrinsic gene, *msr*(C) (Boodaghi Malidareh et al., 2022; Miller et al., 2014). Almost all sequenced *E. faecium* strains in this study, except strain E5‐100, carried the *msr*(C) gene. Although this gene was detected in all strains, some strains did not show phenotypic resistance against quinupristin/dalfopristin. In addition to *E. faecium*, we also detected some *E. faecalis* strains in our samples. *E. faecalis* is intrinsically resistant to quinupristin/dalfopristin due to the chromosomal gene *lsa* (Stedman et al., 2020). However, all of the *E. faecalis* strains obtained in this study were sensitive to quinupristin/dalfopristin. The lack of phenotypic resistance in these strains carrying ARGs might be due to low or no expression of these genes. Further studies on transcriptomics and/or proteomics are needed to confirm these findings.

Apart from resistance against quinupristin/dalfopristin, ciprofloxacin resistance was also observed in *E. faecium* strains from both influents (4.2%) and treated effluents (3.2%). This is similar to the observed prevalence of ciprofloxacin resistance in *E. faecium* strains (5.9%) from cecal samples from fattening pigs in Norway in 2021 (NORM/NORM‐VET, 2022). High‐level ciprofloxacin resistance in *E. faecium* has previously been linked to *gyrA* and *parC* mutations (Rathnayake et al., 2012; Werner et al., 2010). We detected mutations in both *gyrA* (p.S83Y) and *parC* (p.S80I) in strains showing high ciprofloxacin resistance (MIC > 16 mg/L), thus explaining the observed resistance against ciprofloxacin. Furthermore, reduced susceptibility (MIC > ECOFF) against several antibiotics, including erythromycin and tetracycline, was detected in some of our strains. For erythromycin, the reduced susceptibility in *E. faecium* strains obtained from the influent samples (30.6%) was higher compared to the effluent samples (24.9%). In contrast, the reported reduced susceptibility to erythromycin from the veterinary setting in Norway was only 2.9% (NORM/NORM‐VET, 2022). Additionally, higher reduced susceptibility to tetracycline was observed in *E. faecium* strains from the effluent samples (8.6%) compared to strains from the influent samples (6.2%). This is much lower than the reported reduced susceptibility to tetracycline (33%) in *E. faecium* strains from pigs in Norway (NORM/NORM‐VET, 2022). We detected the presence of *erm*(B), *erm*(T), *tet*(M), and *tet*(L) in several sequenced *Enterococcus* spp. strains, thus explaining the observed phenotypic resistance against the respective antibiotics (Aarestrup et al., 2000; Cauwerts et al., 2007; Chajęcka‐Wierzchowska et al., 2016). These ARGs have also been shown to be prevalent in *Enterococcus* spp. strains from sewage from different countries like South Africa (Mbanga et al., 2021), Tunisia (Ben Said et al., 2015), and Canada (Zaheer et al., 2020).

Clinically relevant *E. faecium* belonging to CC17 can be characterized based on resistance against ampicillin, quinolones, and sometimes high‐level resistance against gentamicin (Leavis, Bonten, et al., 2006; Leavis, Willems, et al., 2006; Quiñones et al., 2018). We detected two MDR strains (E4‐112 and E5‐100) with resistance against ampicillin, ciprofloxacin, and gentamicin (high‐level) in the treated effluent in this study. Additionally, reduced susceptibility to erythromycin was also observed in one of the MDR strains (E5‐100). Whole genome phylogeny showed that the MDR strains belonged to the clinically relevant CC17 clade A1 (Lee et al., 2019), which represents a high‐risk epidemic clonal linage of *E. faecium* (Leavis, Bonten, et al., 2006) known for causing severe morbidity and mortality (Lee et al., 2019). Although no β‐lactamase was detected in our strains, several penicillin‐binding proteins (PBPs) were present. The high resistance against ampicillin observed in our study may be due to the presence of several PBPs and/or increased production of PBP5 (Jureen et al., 2003; Sifaoui et al., 2001). Although this is highly likely, further studies are needed to confirm the expression of PBPs in our strains. Apart from carrying several ARGs, the strains also carried important virulence factors involved in adherence (*ebpBC*, *srtC*, *ecbA*, *esp*, and *pavA*) (Leclercq et al., 2013), antiphagocytosis (*cpsB/cdsA* and *cpsC*), biofilm formation (*fsrA*) (Leclercq et al., 2013), immune evasion (*cps2K* and *epsE*), protease (*tig/ropA*), and serum resistance/immune evasion (LPS rfb locus from *Klebsiella*), suggesting pathogenic potential of these MDR strains. *E. faecium* CC17 can persist in aquatic environments polluted by sewage (Leclercq et al., 2013), including marine environments (Vignaroli et al., 2013). Hence, there is a risk that these MDR strains can persist in the marine environments in Norway (Cherak et al., 2022). Our study thus demonstrates that clinically important MDR strains of *E. faecium* carrying several ARGs and virulence genes are entering the marine environment in Norway through treated effluents.

## 5 CONCLUSIONS

All the *Enterococcus* spp. strains in our study were sensitive to last‐resort antibiotics like linezolid, vancomycin, and tigecycline, which is in accordance with the clinical prevalence in Norway (NORM/NORM‐VET, 2022). Our findings along with clinical prevalence and studies from the animal sector show that resistance against last‐resort antibiotics in *E. faecium* in Norway, in general, is very low (NORM/NORM‐VET, 2022). Although true, we did detect clinically important ARGs and clinically relevant MDR strains in both influent and treated effluent entering the receiving marine environment. Our study highlights the importance of sewage‐based surveillance to monitor and understand the resistance in pathogens within the population as well as demonstrates that treated sewage is a source of clinically important MDR pathogens entering the receiving marine environment.

## AUTHOR CONTRIBUTIONS

**Vera Radisic**: Methodology (lead); investigation (equal); validation (equal); visualization (lead); formal analysis (equal); bioinformatic analysis (equal); analysis of data (lead); writing—original draft (lead); writing—review and editing (lead). **Didrik H. Grevskott**: Methodology (supporting); visualization (supporting); analysis of data (supporting); writing—review and editing (supporting). **Nadja Junghardt**: Methodology (supporting); investigation (supporting); bioinformatic analysis (equal); writing—review and editing (supporting). **Lise Øvreås**: Critical inputs (supporting); writing—review and editing (supporting). **Nachiket P. Marathe**: Funding acquisition (lead); project administration (lead); supervision (lead); conceptualization (lead); methodology (supporting); investigation (equal); supervision (lead); validation (equal); visualization (supporting); writing—review and editing (supporting).

## CONFLICT OF INTEREST STATEMENT

None declared.

## ETHICS STATEMENT

None required.

## Supporting information

Supporting information.

Supporting information.

Supporting information.

Supporting information.

Supporting information.

## Data Availability

All data are provided in full in the results section and supporting information of this paper, except the assembled genome sequences which are available in GenBank under the following accession numbers: JAPMLK000000000, JAPMLJ000000000, JAPRGL000000000, JAPRGK000000000, JAPRGJ000000000, JAPRGI000000000, JAPRGH000000000, JAPRGG000000000, JAPMLI000000000, JAPRGF000000000, JAPRGE000000000, JAPMLH000000000, JAPRGD000000000, JAPRGC000000000, JAPMLG000000000, JAPRGB000000000, JAPRGA000000000, JAPRFZ000000000, JAPRFY000000000, JAPRFX000000000, JAPRFW000000000, JAPRFV000000000, JAPRFU000000000, JAPRFT000000000, and JAPRFS000000000.
